# Plant organ cultures as masked mycotoxin biofactories: Deciphering the fate of zearalenone in micropropagated durum wheat roots and leaves

**DOI:** 10.1371/journal.pone.0187247

**Published:** 2017-11-16

**Authors:** Laura Righetti, Enrico Rolli, Gianni Galaverna, Michele Suman, Renato Bruni, Chiara Dall’Asta

**Affiliations:** 1 Department of Food and Drug, University of Parma, Parma, Italy; 2 Deparment of Chemistry, Life Sciences and Environmental Sustainability, University of Parma, Parma, Italy; 3 Barilla G.R. F.lli SpA, Advanced Laboratory Research, Parma, Italy; Universita degli Studi di Pisa, ITALY

## Abstract

“Masked mycotoxins” *senso strictu* are conjugates of mycotoxins resulting from metabolic pathways activated by the interplay between pathogenic fungi and infected plants. Zearalenone, an estrogenic mycotoxin produced by *Fusarium* spp, was the first masked mycotoxin ever described in the literature, but its biotransformation has been studied to a lesser extent if compared to other compounds such as deoxynivalenol. We presented herein the first application of organ and tissue culture techniques to study the metabolic fate of zearalenone in durum wheat, using an untargeted HR-LCMS approach. A complete, quick absorption of zearalenone by uninfected plant organs was noticed, and its biotransformation into a large spectrum of phase I and phase II metabolites has been depicted. Therefore, wheat organ tissue cultures can be effectively used as a biocatalytic tool for the production of masked mycotoxins, as well as a replicable model for the investigation of the interplay between mycotoxins and wheat physiology.

## Introduction

Modified mycotoxins have recently become a prominent issue in food safety research and risk assessment, due to the increasing awareness of possible toxic effects related to their (co)occurrence in food. In particular, “masked mycotoxins” *senso strictu* are those conjugates resulting from metabolic pathways activated by the interplay between pathogenic fungi and infected plants [1]. Several masked mycotoxins have been described in cereals so far, including derivatives of deoxynivalenol (DON), nivalenol, T-2 and HT-2, alternariol and alternariol methyl ether [1, 2]. Being lipophilic, these compounds may cross the membrane and distribute into plant cell organelles, where they both exert their toxicity and are exposed to enzymatic pools. Biotransformations are believed to be part of the plant detoxification system; xenobiotics carrying hydroxyl groups can be conjugated to a sugar and further processed by addition of a malonyl, hexose or pentose moiety to facilitate translocation, compartmentation and storage, while further hydroxyls may be directly added [3]. Mycotoxin conjugates are not monitored in routine food control and their direct toxicity may be uncertain. Upon ingestion the parent form may be released in the digestive tract and absorbed, thereby the total exposure to the original mycotoxin in both humans and animals [4] may be increased.

**Fig 1:**
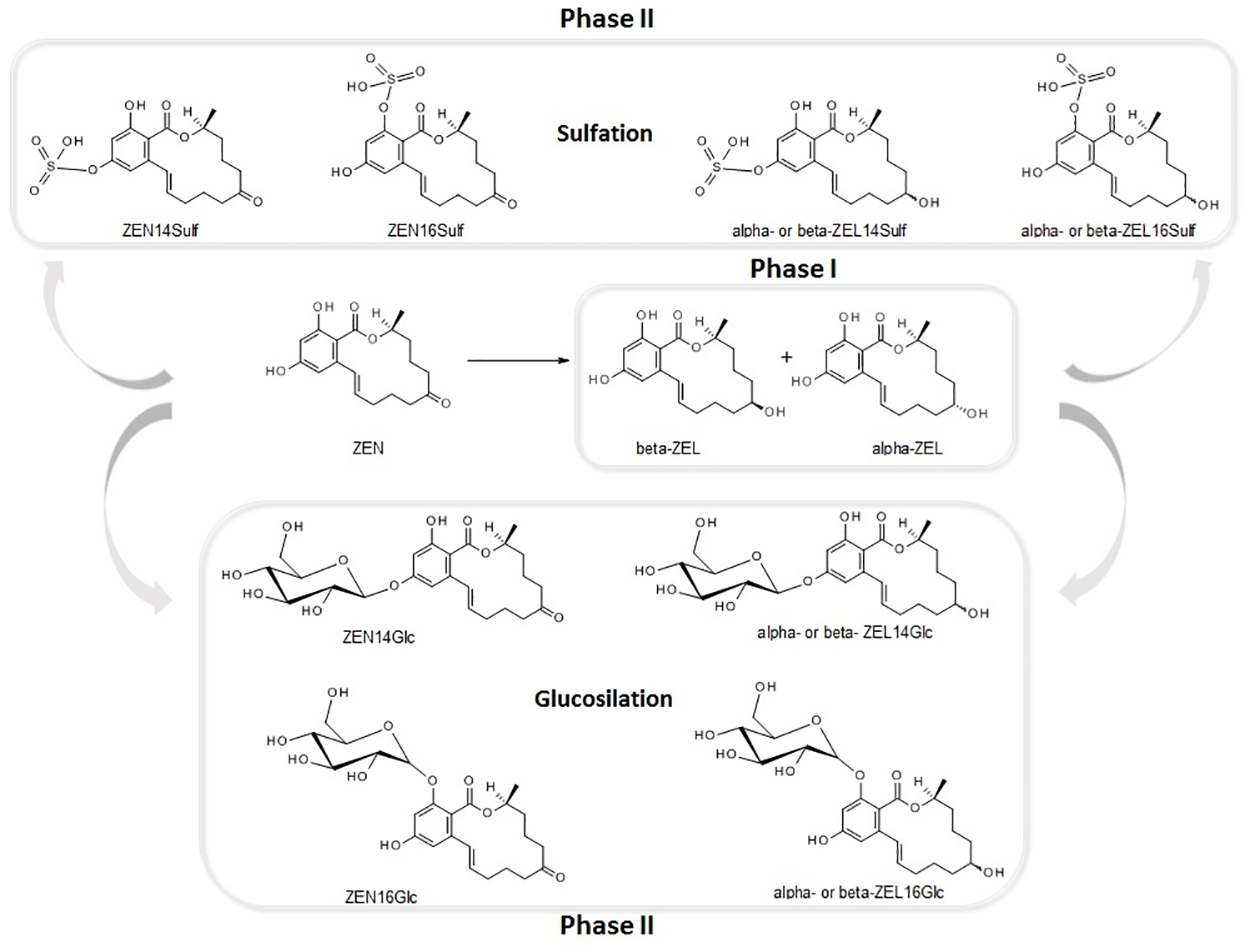
Main known phase I and phase II metabolites of ZEN.

Zearalenone (ZEN, Fig 1) is produced by several *Fusarium* species found in both rhizosphere and phyllosphere of healthy cereal plants, where they act as soil saprophytes or behave as parasites or pathogens in both pre- and post-harvest stages [5, 6]. When occurring, the infection is actuated by colonizing parenchymatous and phloematic tissues, with mycelia penetration from both the rhizoplane and stomata and by means of specialized infection cushions. Such process is accompanied by necrosis and by the biosynthesis of a wide array of toxic fungal secondary metabolites including ZEN. Contrarily to most trichothecenes, ZEN is produced by *Fusarium* strains also during non-pathogenic growth. Although not exerting severe acute toxicity in plants and animals, ZEN is known for a strong estrogenic and hormone-like activity [7, 8]. In living organisms ZEN undergoes reductive phase I metabolism with the formation of α- and β-zearalenol (ZELs), of the saturated form zearalanone (ZAN) and its reduced metabolites α- and β-zearalanol (ZALs) [9], which may possess an even higher endocrine disrupting behavior than their parent compound [10–12]. Although ZEN largely occurs as *trans*-isomer, it has been reported that *cis*-ZEN may be formed upon light exposure and in water, in presence of some ionic species [13]. The *cis*-isomer is usually overlooked in food analysis, but its presence in edible plant matrices has been reported and in mammals its metabolites have comparable estrogenicity as *trans*-ZEN [14–16]. In infected cereals, ZEN may undergo conjugation through glycosylation; zearalenone-14-glucoside (ZEN14Glc) is its most known masked form [1]. Despite being the first masked mycotoxin ever described in the literature [17], its biotransformation has been studied to a lesser extent if compared to major trichothecenes such as deoxynivalenol (DON), and the isomer ZEN16Glc was only recently elucidated [18]. Also sulfation products have been reported in naturally infected cereals, but their structure is still to be univocally elucidated. It’s not yet clear if sulfates should be considered as plant or fungal metabolites of ZEN, given the fact that many *Fusarium* species are autonomously capable of their biosynthesis [19, 20]. Recent reports highlighted the (co)occurrence of ZEN, its conjugated forms, its phase I metabolites α- and β-ZEL, and the conjugated forms thereof, in naturally infected cereals [21].

These findings are in agreement with the results obtained using uninfected *A*. *thaliana* as model system [22]. Healthy plants are known for their capability to biotransform a wide range of both natural and man-made xenobiotics and few papers have suggested that also mycotoxins may be absorbed by asymptomatic crops including rice, lettuce, sugarcane, asparagus and peanut [23–28].

To evaluate the physiological response of plants to xenobiotics and in consideration of lower cost and more controlled conditions, *in vitro* systems such as cell cultures are often preferred to fields trials and greenhouse experiments, despite their obvious distance from natural conditions [29].

An investigation of the interplay between ZEN and wheat by means of model systems based on organ cultures may represent a fascinating but yet scarcely explored frontier to improve our understanding of masked mycotoxins [30]. The biocatalytic potential of healthy plants and cultured cells on mycotoxins has been evaluated both recently and in the past, but ZEN has been mostly overlooked. If compared to trichothecenes like DON and T-2, our knowledge on *in planta* biotransformations of ZEN by wheat is limited. Furthermore, the literature is focused on a very limited range of conjugated compounds, not taking full advantage of modern analytical tools [31, 32]. For instance, to comprehensively study the metabolism of mycotoxins *in planta*, stable isotope labelling (SIL) has been successfully proposed for the elucidation of T-2 biotransformation in wheat [33–35]. Nonetheless, such approach requires dedicated software for signal comparison and deconvolution, and the use of isotope-labelled standards in high amount.

As a major advantage over targeted methods, untargeted metabolomics approaches based on mass-spectrometry have the potential to discover unknown biotransformation products originating from specific xenobiotics when limited previous knowledge is available [36]. A targeted-untargeted metabolomics approach was therefore selected to investigate both phase I and II biotransformation of ZEN in healthy roots and leaves of micropropagated *Triticum durum* Desf., with the goal to assess their potential as masked mycotoxins biofactories and to evaluate the physiological response of the plant metabolism to ZEN exposure.

## Materials and methods

### Chemicals and reagents

Analytical standards of ZEN (100 μg mL^-1^ in acetonitrile), α-ZEL (solution in acetonitrile 10 μg mL^-1^) and β-ZEL (solution in acetonitrile 10 μg mL^-1^) were obtained from Sigma-Aldrich (Taufkirchen, Germany). Zearalenone-14-glucoside (ZEN14Glc) was synthesised and purified in our laboratory [4], as well as *cis*-zearalenone [14]. Zearalenone-16-glucoside (ZEN16Glc) was kindly provided by Prof. Franz Berthiller (IFA-Tulln, University of Natural Resources and Life Science, Vienna). HPLC-grade methanol, acetonitrile and acetic acid, as well as dimethylsulfoxide (DMSO) were purchased from Sigma-Aldrich (Taufkirchen, Germany); bidistilled water was obtained using a Milli-Q System (Millipore, Bedford, MA, USA). MS-grade formic acid from Fisher Chemical (Thermo Fisher Scientific Inc., San Jose, CA, USA) and ammonium acetate (Fluka, Chemika-Biochemika, Basil, Switzerland) were also used.

### Culture medium and ZEN solutions

Cultures were carried out on MS medium, added with 3% (w/v) sucrose. The medium was solidified with 0.8% (w/v) phyto agar and pH was adjusted to 5.8 with 0.1 M NaOH before autoclaving at 121°C for 20 minutes. Liquid cultures were prepared as above without agar. All the experiments were carried out in triplicate and repeated three times.

### Plant material

Two commercial durum wheat (*Triticum durum* Desf.) varieties, namely Kofa and Svevo, were selected for their previous different FHB resistance and are early-flowering genotypes well adapted to the Mediterranean climate [37].

Kofa, is a Southwestern United States cv. released by Western Plant Breeders (Arizona) obtained from a population based on multiple parents (dicoccum alpha pop-85 S-1) mainly related to the American and CIMMYT germplasm, with the inclusion of emmer accessions.

Svevo, an Italian cv. released by Società Produttori Sementi, has been obtained from the cross between a CIMMYT line (pedigree rok/fg//stil/3/dur1/4/sapi/teal//hui) related to the cv. Yavaros, genetic background (Jori/Anhinga//Flamingo), and the cv. Zenit originating from a cross between Italian and American accessions (Valriccardo/Vic).

#### Micropropagation

Caryopsis were soaked 70% (v/v) ethanol for five minutes, rinsed 3 times in sterile distilled water. After rinsing, seeds were kept in the dark in distilled water for 5 hours at 28°C. Surface disinfection was performed with 2.5% (v/v) sodium hypochlorite for 25 min, followed by six washes with sterilized distilled water. The sterilized caryopsis were cultured individually in glass culture tubes containing about 15 ml of ¼ strength MS medium. Cultures were maintained in a growth chamber at 25° ± 1°C with a 16 h photoperiod under fluorescent tubes at a light intensity of 27 μmol m^–2^ s^–1^. One week after germination, seedlings grown above 5 cm in length were selected and segments of 10 mm containing apical meristems and leafs were obtained by transverse cuts with a scalpel blade. Explants were cultured on shoot multiplication medium (SM), containing MS basal salt medium, added with 8.88 μM N6-benzyladenine (BAP) and 2.2 μM 2,4-dichlorophenoxyacetic acid (2,4-D). Multiple shoot clumps arising from the shoot apices were divided and subcultured in MS every 3 weeks.

#### Root culture

For root induction, shoots were cultured on agarized MS medium *hormone free*. After 4 weeks, roots were excised and inoculated in liquid MS medium (50 ml) supplemented with 1 μM IBA in glass conical flasks (150 ml); Cultures were kept in the dark under continuous agitation at 100 rpm in an orbital shaker and maintained in climatic chamber for 4 wk. To improve root growth, 1 μM IBA, as auxin, was added in the roots culture

#### Leaf culture

Leaves (3–5 cm in length) were excised from 3 weeks old plants and placed in 50 ml test tube containing few milliliters of solid MS medium. The leaf base was immersed into the medium, then the tubes were filled with liquid medium (MS) added with 10 μM BAP, sealed and incubated in climatic chamber for two weeks. Cytokinin BAP at 10 μM was present in the leaf culture medium to prevent tissue senescence.

### Sample preparation, ZEN administration, sampling and controls

ZEN was dissolved in an adequate amount of DMSO so that the final concentration of the solvent in culture medium did not exceed the one considered toxic (0.2%) with mycotoxin being at the final concentration of 12.5 μg/L and 100 μg/L. Solutions were sterilized by 0.2-μm filters and dissolved in the liquid medium in leaves-containing tubes and in root-containing flasks. Leaves (approx. 200 mg for each tube), were anchored to the bottom of tubes by immersion of the basal part in a fine layer of solid medium, allowing a constant exposure of the emerging organ in liquid medium. Roots (approx. 600 mg for each flask) were instead suspended in liquid medium and kept in the dark under orbital shaking (90 rpm). Liquid medium without mycotoxin was used in all experiments as a control. To monitor the evolution of its absorption, ZEN presence in liquid media was determined six times in both leaves and roots cultures and in flask containing solely liquid medium at the following intervals: t = 0, t = 12h, t = 1d, t = 7d and t = 14d. At the end of the experiment neither leaves nor roots cultures exposed to 100 μg/l ZEN showed any visible degradation.

### Sample extraction and analysis

Plant samples were freeze dried for 24 h using a laboratory lyophylizator (LIO-5PDGT, 5Pascal s.r.l., Trezzano sul Naviglio, Milano) and then milled.

Plant samples (50 mg of homogenized material) were extracted with a mixture of acetonitrile/water/formic acid (79:20:1, v/v), and stirred for 90 min at 200 strokes/min on a shaker. The extract was centrifuged for 10 min at 14000 rpm at room temperature, then 500 μL of supernatant were evaporated to dryness under nitrogen and finally reconstructed by 500 μL of water/methanol (80:20, v/v) prior to LC-MS analysis.

To monitor the evolution of ZEN and the possible secretion of masked mycotoxins, medium samples were taken (in aseptic condition) from both root and leaf culture at the start of experiment (t0), then after 1, 6, 12, 24 hours, 7 and 14 days and kept refrigerated until analysis. All medium samples were diluted with water/methanol (80:20, v/v) to achieve a final ratio of 1:1 (v/v), vortexed for 1 min and then subjected to LC-MS analysis.

UHPLC Dionex Ultimate 3000 separation system coupled to a Q-ExactiveTM high resolution mass spectrometer (Thermo Scientific, Bremen, Germany) equipped with an electrospray source (ESI) was employed. For the chromatographic separation, a reversed-phase C18 Kinetex column (Phenomenex, Torrance, CA, USA) with 2.10×100 mm and a particle size of 2.6μm heated to 40°C was used. 10 μl of sample extract was injected into the system; the flow rate was 0.4 ml/min. Gradient elution was performed by using 1 mM ammonium acetate in water (eluent A) and methanol (eluent B) both acidified with 0.5% acetic acid. Initial conditions were set at 10% B followed by a linear change to 40% B in 4 min and to 90% B in 16 min. Column was then washed for 2 min with 90% B followed by a reconditioning step for 3 min using initial composition of mobile phases. The total run time was 25 min. The Q-Exactive mass analyzer was operated in the full MS/data dependent MS/MS mode (full MS—dd-MS/MS) at following parameters: sheath and auxiliary gas flow rates 32 and 7 arbitrary units, respectively; spray voltage 3.3 kV; heater temperature 220°C; capillary temperature 250°C, and S-lens RF level 60. Following parameters were used in full MS mode: resolution 70,000 FWHM (defined for m/z 200; 3 Hz), scan range 100–1000 m/z, automatic gain control (AGC) target 3e6, maximum inject time (IT) 200 ms. Parameters for dd-MS/MS mode: intensity threshold 1e4, resolution 17,500 FWHM (defined for m/z 200; 12 Hz), scan range 50 –fragmented mass m/z (m/z +25), AGC target 2e5, maximum IT 50 ms, normalized collision energy (NCE) 35% with ±25% step.

The full identification of ZEN, α-ZEL, and β-ZEL was obtained by comparison with commercial standards. Similarly, *cis*ZEN, ZEN14Sulf, ZEN14Glc, and ZEN16Glc were accurately identified by comparison with authentic standards, obtained by chemical or enzymatic synthesis [4, 8, 18]. For other metabolites, the annotation process involved the following items; (i) the measured accurate mass of [M-H]¯ must fit the theoretical accurate mass with a mass tolerance set at ±5 ppm, (ii) isotopic pattern: the experimental and theoretical isotopic patterns shall correspond, (iii) MS-MS spectra: product ion of intact ZEN (m/z 317.1389) and ZEL (m/z 319.1550) and/or comparison of the fragments obtained with the fragmentation pathway of ZEN or other mycotoxins metabolites formerly found [18, 21, 33–35]. Only in few cases, fragmentation spectra could not be collected, due to parent ion abundance below the threshold. In this case, a tentative annotation based on accurate mass and elemental formula was performed, as already proposed by other authors [33–35].

### Statistical analysis

All statistical analyses were performed using IBM SPSS v.23.0 (SPSS Italia, Bologna, Italy). Data were analysed by Kruskal-Wallis test followed by Duncan post-hoc test (α = 0.05).

## Results

### Qualitative screening of ZEN conjugates

**Fig 2:**
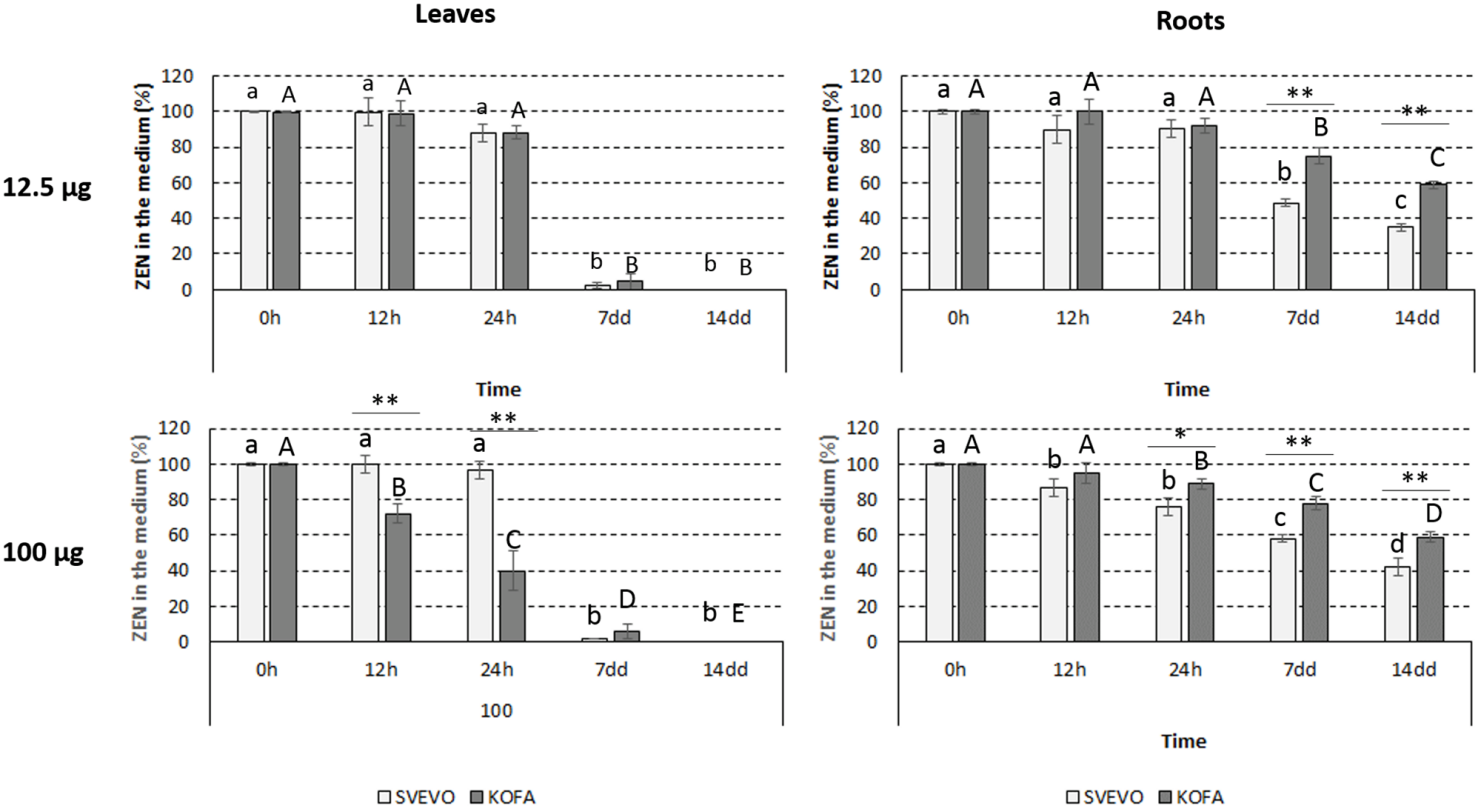
Absorption of ZEN from the growing medium (initial amount: 100 μg). Data are given in terms of residual ZEN% in the medium (n = 4). A) Leaves; B) roots. Statistical significance was performed by non prametric Kruskal-Wallis test (α = 0.05). Different letters indicate significant differences at different time points within the same cultivar. Differences at the same time point between the two cultivars were indicated by an asterisk (*: p < 0.05; **: p < 0.005).

To focus on the effect of plant metabolism under physiological conditions, a qualitative screening was performed by contaminating culture media with calibrated amounts of pure ZEN. Two separate amounts were administered after previous checking the tolerance of cultured leaves and roots during the whole time course of the experiment, in order to avoid concentrations known for causing toxicity in maize root cells and barley seedlings [38]. The final ZEN concentrations in the growing medium were 12.5 and 100 μg/L, respectively. In both Kofa and Svevo cultivars the mycotoxin was quantitatively absorbed after 7 days in leaves, with minor differences between the two administered amounts (Fig 2). When the lower amount was administered, the absorption was faster in Kofa than in Svevo, but differences were leveled up at 100 μg/L. In roots, the absorption was slower and less efficient although comparable at both amounts of administered ZEN. The absorption was more efficient in Svevo than in Kofa, with a final residual amount of ZEN in the medium of about 40% and 60% respectively, at both administered concentration levels.

As xenobiotics may be both modified by intracellular enzymes, by enzymatic pools secreted into the soil or also diffused back in the rhizosphere once biotransformed, and because light and ion exposure may induce ZEN isomerization, the growing media was carefully monitored. Our data show that no known masked mycotoxins were produced or diffused in the growing media, and that no degradation occurred due to chemical and physical agents during the whole experiment, with the sole exception of the expected isomerization from *trans*- to *cis*-ZEN occurring only after 7 days and reaching a 40:60 *trans*:*cis* ratio at 14 days (**Figs A, B, and C in** S1 File).

Previous reports have also hypothesized that ZEN may be in some occasions an endogenous product of plant metabolism, acting as a growth and flowering regulator, in particular during vernalization [39]. To avoid any interference on this regard, controls with untreated plants were set up and resulted negative both at the beginning and at the end of the experiment (**Fig D in** S1 File).

Since few modified forms of ZEN are available as reference compounds (i.e. ZEN14Glc, ZEN16Glc, α- and β-ZEL), we followed an untargeted qualitative approach. A total of 64 chromatographic peaks were obtained, resulting in 10 putative phase I metabolites (Table 1) and 18 putative phase II compounds (Table 2). Of those, 16 feature groups were of sufficient intensity, enabling their structural elucidation by HR-MS/MS. In some cases, different peaks were assigned to one single putative metabolite, in consideration of the possible isomeric forms.

**Table 1:** Phase I metabolites of ZEN annotated from roots and leaves analysis and their qualitative abundance (i.e. N.D: not detected, +: detected with low relative abundance; ++: detected with high relative abundance).

Peak no.	RT	Formula	Detected *m/z* [M-H]¯	Mass error ppm	Putative metabolite	Roots	Leaves
57	10.14	C18H20O5	315.1237	0.34	Dehydro-ZEN^a^	++	N.D.
	12.14	C18H22O5	317.1338	-1.97	ZEN*	++	+
	12.41	C18H22O5	317.1388	-1.75	*cis*-ZEN*	+	++
	11.94	C18H24O5	319.1544	1.50	α-ZEL*	+	++
	10.39	C18H24O5	319.1548	-0.08	β-ZEL*	+	++
37;	8.72;	C18H20O6	331.1185;	-0.45;	Hydroxy-dehydro-ZEN^a^	++	++
46	9.26		331.1185	-0.45			
16;	7.23;	C18H22O6	333.1338;	1.66;	Hydroxy-ZEN or Hydroxy-dehydro-ZEL^a^	+	++
28;	8.09;		333.1338;	1.84;			
38;	8.73;		333.1343;	-0.15;			
45;	9.26;		333.1342;	-0.33;			
54;	9.70;		333.1344;	-0.20;			
58	10.24		333.1340	-0.99			
20;	7.51;	C18H22O6	333.1345;	0.66;	Hydroxy-ZEN or Hydroxy-dehydro-ZEL^a^	++	N.D.
59	10.38		333.1340	-0.81			
56	9.83	C18H24O6	335.1497	-0.15	Hydroxy-ZEL^a^	+	++
26	7.97	C18H24O6	335.1497	-0.15	Hydroxy-ZEL^a^	++	N.D.

* Confirmation with standard by comparison of accurate mass, HRMS/MS and RT.

^a^ Annotation with accurate mass, elemental formula and HRMS/MS spectra.

**Table 2:** Phase II metabolites of ZEN annotated from roots and leaves analysis and their qualitative abundance. (i.e. N.D: not detected, +: detected with low relative abundance; ++: detected with high relative abundance).

Peak no.	RT (min)	Formula	Detected *m/z* [M-H]¯	Mass error (ppm)	Putative metabolite	Roots	Leaves
ZEN-Sulf	7.83	C18H22O8S	397.0964	0.44		++	+
7;	6.22;	C18H24O8S	399.1120;	0.29; 0.67	α- or β-ZEL-Sulf^a^	++	N.D.
24	7.93		399.1121				
ZEN-16-Glc*	6.30	C24H32O10	479.1915	-1.48		+	++
ZEN-14-Glc*	8.46	C24H32O10	479.1917	-1.04		++	+
50	9.32	C24H34O11	479.202	0.52	Hydroxy-ZEL-Glc^a^	N.D.	++
1;	5.46;	C24H34O10	481.2076;	1.75;	ZEL-Glc^a^	+	++
4;	5.97;		481.2071;	-0.89;			
10;	6.70;		481.2074;	-1.53;			
33	8.38		481.2078	-1.60			
15;	7.18;	C24H32O11	495.1860;	-0.09;	Hydroxy-ZEN-Glc^a^	N.D.	++
21;	7.63;		495.1857;	-0.66;			
40	8.97;		495.1857;	-0.72;			
5;	6.01;		495.1871;	2.10;		++	N.D.
11	6.76;		495.1864;	0.69;			
2	5.47		495.1864;	0.81		++	+
19;	7.45;	C27H34O13	567.2074;	-1.61;	ZEN-MalGlc^a^	++	+
49	9.31;		565.1918;	-1.47;			
48;	9.29;		567.2075;	-1.40;		N.D.	++
52;	9.59;		567.2077;	-0.97;			
55	9.75;		565.1921;	-0.92;			
8;	6.30;	C29H40O14	611.2324;	-0.75;	ZEN-HexPent^a^	N.D.	++
34	8.47		611.2330	-0.56			
6;	6.18;	C30H42O15	641.2446;	-0.72;	ZEN-di-Glc^a^	++	N.D.
17	7.41		641.2445	-0.81			
3;	5.61;		641.2452;	0.22;		N.D.	++
32	8.22		641.2456	0.80			
13;	7.13;	C30H44O15	643.2586;	-1.51;	ZEL-di-Glc^a^	++	N.D.
18	7.42		643.2606	-0.29			
31	8.19		643.2603	-0.56		N.D.	++
42;	9.01;	C33H44O18	727.2457;	0.41;	ZEN-Mal-di-Glc^b^	+	++
51	9.45		727.2454	-0.01			
41;	8.97;	C33H46O18	729.2611;	1.46;	ZEL-Mal-di-Glc^b^	N.D.	++
47	9.27		729.2610	1.38			
23	7.91	C36H52O20	803.2984	0.60	ZEN-tri-Glc^a^	++	N.D.
44;	9.2; 9.6	C36H46O21	813.2452;	-0.79;	ZEN-di-Mal-di-Glc^b^	+	++
53			813.2464	0.71			
9;	6.41;	C36H48O21	815.2603;	-0.22;	ZEL-di-Mal-di-Glc^b^	N.D.	++
30;	8.18;		815.2606;	0.30;			
36;	8.67;		815.2612;	1.04;			
43	9.17		815.2606	0.22			
25;	7.94;	C39H54O24	889.2977;	0.64;	ZEN-di-Mal-tri-Glc^b^	+	++
29	8.09		889.2980	0.91			
12;	7.02;	C39H56O23	891.3123;	-0.54;	ZEL-di-Mal-tri-Glc^b^	N.D.	++
14;	7.16;		891.3129;	0.14;			
22;	7.89		891.3134;	0.69;			
39	8.65		891.3137	0.96			

* Confirmation with standard by comparison of accurate mass, HRMS/MS and RT.

^a^ Annotation with accurate mass, elemental formula and HRMS/MS spectra.

^b^ Annotation with accurate mass and elemental formula.

**Fig 3:**
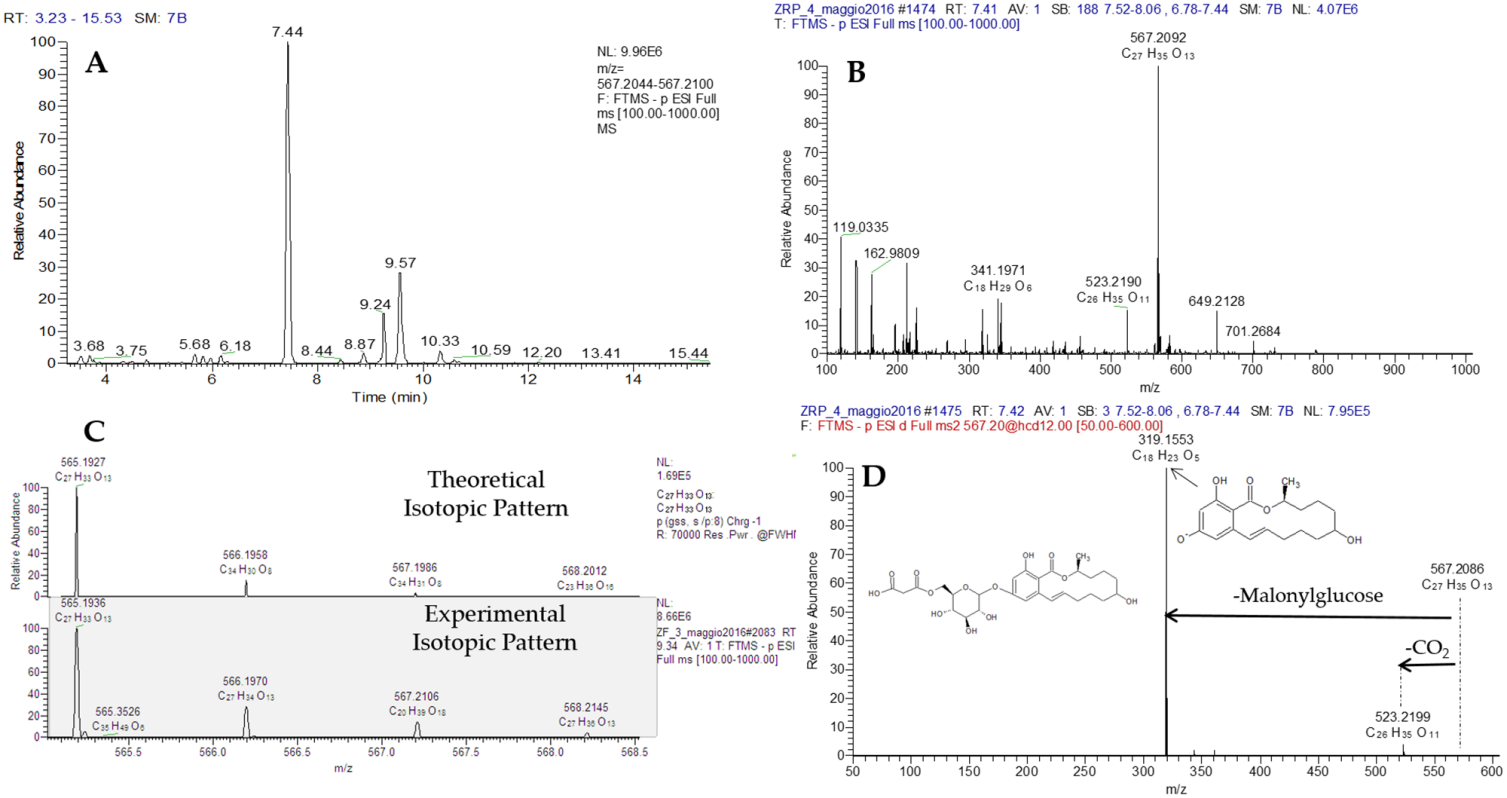
Identification steps used for the putative assignement of ZEN-MalGlc. UHPLC-Q-Exactive full scan (A) extracted ion chromatogram (resolving power 70,000 FWHM, extraction window 5 ppm) and (B) molecular formula assigment of parent ion. Theoretical and experimental isotopic pattern (C) and (D) high resolution fragmentation pathways, obtained by using DDA acquisition, of ZEL-MalGlc.

The identification process used for metabolite putative assignment is depicted in Fig 3, using ZEL-MalGlc as example. From the full scan, the extracted ion chromatogram of ZEL-MalGlc is selected (A); then the parent ion molecular formula (B) is assigned and theoretical and experimental isotopic pattern (C) are compared in order to reduce the number of possible candidates. In the last step, the HR fragmentation pathway (D), obtained by using data dependent acquisition (DDA), facilitate compound identification. The same process was used for all the annotated compounds.

#### Phase I metabolites

**Fig 4:**
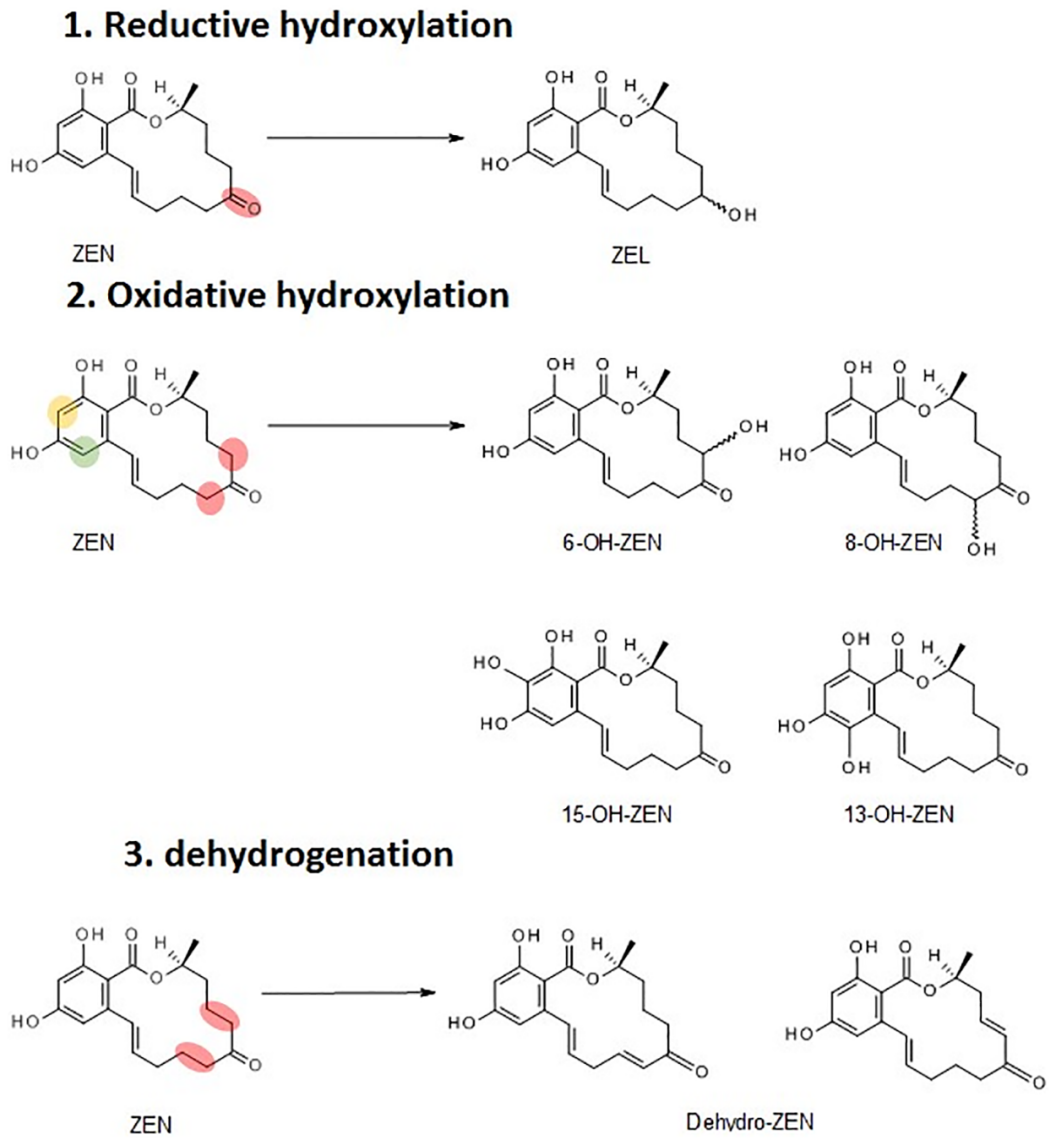
Main possible phase I metabolites of zearalenone according to Site of Metabolism (SOM) prediction.

Many hydroxylated forms were identified, but few differences were noticed between the tested cultivars. Those masked mycotoxins are ascribable not only to the reduction of the keto group, giving rise to the formation of α- and β-ZEL, but also to the hydroxylation on both the aromatic and the macrocyclic ring. Possible structures are reported in Fig 4, as obtained by Sites of Metabolism (SOM) prediction analysis.

**Fig 5:**
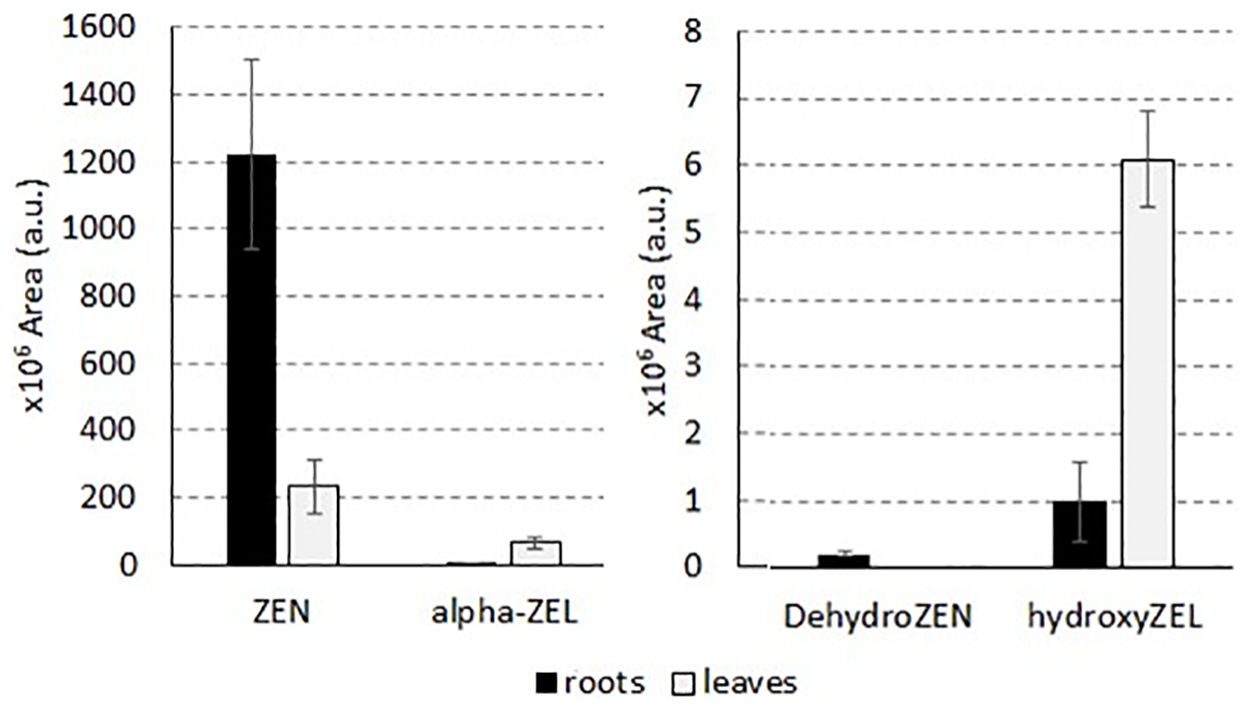
Compounds found to significantly differ in terms of area in durum wheat root and leaf cultures (Kruskal-Wallis test, α = 0.05).

According to the collected results, the formation of phase I metabolites seems to be organ-related, but in consideration of the lack of commercial standards, a quantification was not possible. However, comparing signals obtained for ZEN and other standard compounds in roots and leaves, matrix-related effects were not observed. Therefore, although matrix-related bias cannot be excluded for novel metabolites, a statistical comparison based on chromatographic area of the metabolite profile found in roots and leaves was performed. Analysis pointed out the identification of those compounds able to discriminate between the two organs, namely ZEN, dehydro-ZEN, α-ZEL, and hydroxy-ZEL, as reported in Fig 5.

No statistical difference was identified between Kofa and Svevo plants. In particular, ZEN and dehydro-ZEN were found to be more abundant in roots than in leaves, while signals related to α-ZEL and hydroxyl-ZEL were higher in leaves. Targeted monitoring of ZEN, ZEN14Glc, ZEN16Glc, α- and β-ZEL returned comparable results in terms of amount detected in Svevo and Kofa *cv*. (p = 0.794), while organ related differences were found in both cultivars (p = 0.027). Therefore, results presented in Tables, Results and Discussion sections were reported considering cultivars as biological replicates but keeping separate leaves and roots.

#### Phase II metabolites

ZEN14Glc, ZEN16Glc, and ZEN14Sulf were identified in both roots and leaves, as presented in Table 2. Monoglycosyl and diglycosyl- conjugates of phase I metabolites were identified as well, but due to the lack of suitable standards, it was not possible to discriminate between the isomeric forms. Mono- and di-malonyl conjugates of ZEN and ZELs were identified as well (**Figs E, F and G in** S1 File). Although occurring in different isomeric forms, their formation seemed to be more abundant in leaves then in roots. Contrarily to phase I metabolites, the accumulation of conjugated compounds shared between both organs did not produce a statistical difference neither between leaves and roots, nor between Kofa and Svevo plants. However, a striking difference emerged for those isomers that are abundant in leaves and absent in roots and vice versa. In particular, isomers related to ZEL-Mal-diGlc, Hydroxy-ZENGlc and ZELdiGlc showed an inverted distribution.

## Discussion

Although the conditions applied within this study do not represent the complexity of an actual environment, the direct exposure to ZEN instead of fungal inoculation allows to define the full biocatalytic potential of phase I and phase II enzymatic pools in durum wheat. This cannot be obtained upon infection, because of the cross-talk between the plant and the pathogen. Upon interaction, local necrosis as well as the different integrity of the involved tissues, may significantly modulate plant metabolism not allowing a discrimination between the plant and the fungi metabolism. Masked mycotoxins here reported should be therefore fully considered as unique products of plant metabolism in normal growing conditions, as already reported in previous studies [4, 34, 40]. Leaves showed a quicker absorption, likely ascribable to a different tissue organization; the presence of a highly lipophilic cuticular layer on wheat leaf epidermis may induce at first a sequestration of ZEN, which enters in the plant tissue only in a second time. On the contrary, the less waxy surface of roots may induce a slower process, which is confirmed by the higher amount of unmodified ZEN found in this organ. This phenomenon is known for other xenobiotics, where root hairs and root cortex usually accumulate higher amounts than stele cells [41]. The formation of modified forms of ZEN in plants has been addressed in the past only using maize cells suspension cultures and *Arabidopsis thaliana* [22, 42] The former study showed that, upon treatment with radiolabelled ^14^C-ZEN, more than 50% of the initial radioactivity was incorporated as insoluble residue in maize cell suspension cultures. Later, the use of *Arabidopsis* as model plant allowed the elucidation of the metabolic transformation of ZEN into its phase I and phase II modified forms [22]. In particular, the authors identified 17 compounds, among them α- and β-ZEL, ZEN14Glc, ZEN14Sulf, and the glycosidic conjugates of α- and β-ZEL, some of them apparently released in the culture media. In addition, several di-hexosides, hexose-pentosides and malonylglycosides of ZEN, α- and β-ZEL were reported for the first time. Given the histological and biochemical differences between mono- and dicotyledons, data acquired on *Arabidopsis* may not be automatically transferred to those crops preeminently involved in ZEN-related food safety issues, i.e. cereals [43]. A noticeable difference in our case is the complete lack of masked mycotoxins in the growing media during the whole course of the experiment in both leaves and roots, whereas both in barley and *Arabidopsis* seedlings such phenomenon was reported, albeit to a minor extent [22, 44]. This different behavior may be related to plant-specific differences, to the higher levels of ZEN exposure in previous experiments or, more likely, to the peculiar conditions of *in vitro* organ culture (for instance, the lack of a transpiration stream, the limited interplay between the plant and the root microbiota and the different medium composition).

In this study, we used organ cultures of roots and leaves obtained from two micropropaged varieties of durum wheat. While organ-specific differences in the formation of ZEN metabolites were observed, the biotransformation of ZEN did not seem to be cultivar-specific, albeit the minimal pool may not be conclusive on this regard. Contrarily, slight differences were previously noticed between cultivars in barley-mediated biotranformation of ZEN into ZEN16Glc [18]. Our findings are in contrast from what reported for the same wheat varieties under greenhouse experiments performed with DON, when cultivar-related differences were observed [37]. Since DON is directly involved in Fusarium Head Blight (FHB) pathogenesis, its biotransformation to the less toxic DON3Glc should be regarded as a mechanism of resistance towards FHB in wheat [45]. On the contrary, a direct involvement in pathogenesis and/or in wheat resistance/susceptibility has not been reported for zearalenone. At the same time, its putative role in plant physiology as a possible auxin-like substance may allow a less intense detoxification and a quicker systematic distribution in both radical and foliar tissues and therefore a potential bioaccumulation in plants.

The content of untrasformed ZEN in roots was higher than in leaves. This may be in part related to the different tissue organization between these organs. Roots have a large amount of non-filtering parenchimatous tissue in the cortical cylinder in which water diffuse via apoplastic route, therefore acting simply as a passive water container in which ZEN may remain dissolved and not actively exposed to cytoplasmatic enzymes.

Relevant amounts of *cis*-ZEN were detected in both leaves and roots, albeit to a lower extent in the latter. As roots were grown in the dark and leaves under a calibrated photoperiodic illumination to simulate actual growth conditions, such behavior is in partial agreement with the hypothesis of a light-mediated isomerization. We cannot exclude that at least a part of the isomerization process may undergo inside the plant tissues, likely as a consequence of a high concentration of ionic species. It was also noticed that *trans*- to *cis*-ZEN conversion occurred in the medium only after 7 days, and this should be therefore carefully considered in future investigations, in particular when the exposure of plants to ZEN is shorter. Notably, *cis*-ZEN followed the same metabolic pathway as *trans*-ZEN, with the formation of a large number of conjugates.

According to our findings, reductive and oxidative hydroxylation, followed by glycosylation and malonyl-conjugation, are major biotransformation pathways of ZEN as response of wheat detoxification also when a *Fusarium* infection is not occurring. Sulfation was identified as well, although at a minor extent. It must be underlined that sulfation seems to be a major pathway in microbes and animals, while it has been described as minor detoxification route in plants [46].

Overall, the modification pathways identified within this study are consistent with those reported in *A*. *thaliana* [22], an evidence that if confirmed may suggest a limited intra and interspecific variability between detoxification pathways of ZEN in plants.

Notably, ZEN is biotransformed into its reduced forms α- and β-ZEL, and both of them may undergo further glycosylation or sulfation. This is a relevant observation, since recent studies showed that the possible occurrence of α-ZEL and its conjugates in food may represent a matter of toxicological concern [19, 46]. In agreement with our findings, the formation of α- or β-ZEL conjugates in infected grains have been recently reported also by Nathaniel et al. [21]. However, the authors did not reported the occurrence of phase I metabolites under field conditions, probably because conjugation pathways are strongly activated under these conditions, to allow the quick detoxification of mycotoxins through compartmentalization. This is in agreement with a quicker metabolic response in field, due to the pathogenic state of the plant under fungal infection. On the other hand, the use of HR-MS on extracts from model plants clearly support the detection of compounds at trace level. Besides already known phase I metabolites such as α-ZEL and β-ZEL, several hydroxylated forms have been observed within this study. Although HR-MS spectra did not allow the univocal structure elucidation, the preferential formation of several forms could be supported by Site of Metabolism (SOM) methods [47]. Studies performed in animals have already depicted the formation via oxidative hydroxylation of 6-OH- and 8-OH-ZEN hydroxyl forms [48], and of 13-OH- and 15-OH- catechol forms [49]. According to our data, isomeric and isobaric forms corresponding to dehydrogenated and hydroxylated metabolites may be formed as well (i.e. peak at t_R_10.47 min, and peaks at t_R_ 37 min and 46 min, respectively). Following SOM prediction, the main sites involved into phase I enzymatic biotransformation of ZEN are summarized in Fig 4.

The possible formation of hydroxylated forms of ZEN in healthy plants opens an important issue of concern, as these metabolites may be comparable or even more active than the parent compound towards the estrogen receptors in mammals [46]. It is known, in fact, that α-ZEL is much more active than ZEN towards ERα, as reported from *in vitro* [10, 11] and *in vivo* [12] studies. Recently, Ehrlich et al. evaluated the possible estrogenic activity of 15-OH-, 6-OH-, and 8-OH-ZEN using computational methods [50].

According to the data collected, endogenous durum wheat enzymes are capable to biotransform ZEN into a wide spectrum of phase I metabolites. These compounds may follow the conjugation pathways already in use for other biotic and man-made xenobiotics [51]. In particular, glycosylation, malonyl-conjugation, and sulfation seem to be the major routes, originating a plethora of stereo- and regioisomers. For most of them, however, data on occurrence and toxicity cannot be collected due to the lack of proper reference compounds and to the cumbersome and demanding synthetic strategies needed to obtain pure compounds. In this context, wheat organ cultures may be successfully exploited as masked mycotoxins biofactories. Leaves can uptake almost quantitatively ZEN from the growing medium after 7 days, and both leaves and roots biotransform it into a large spectrum of metabolites, following a cost-effective, reproducible and green procedure. Taking into consideration that, in our assay, 100 μg of ZEN were uptaken almost quantitatively into 200 mg of leaf tissue, the isolation and purification of a wide array of modified compounds could be foreseeable. Therefore, following a proper scale up, this approach may be exploited for batch production of ZEN masked mycotoxins. Data reported herein suggest that an organ-related biotrasformation may occur for phase II metabolites as noticed for hydroZENGlc, ZENdiGlc isomers and for ZENtriGlc or ZENMal-diGlc. If confirmed, this will enable the fine-tuning of organ-related strategies for the biotechnological production of masked mycotoxins. Furthermore, the recourse to cultured roots and leaves may represent at the same time an useful tool to evaluate the purported endogenous plant-hormone role of zearalenone that some authors have suggested in the past [39].

## Conclusion

The present study represents the first application of organ and tissue culture techniques to study the metabolic fate of zearalenone in durum wheat. Using an untargeted HR-LCMS approach, a complete, quick absorption of up to 100 μg/L of ZEN by uninfected plant organs was noticed and its biotransformation into a large spectrum of phase I and phase II metabolites has been depicted. Therefore, wheat organ tissue cultures can be effectively used as replicable model for the investigation of masked mycotoxin formation. The same technology, however, has the potential to be applied as a biocatalytic tool for the production of masked mycotoxins and for the investigation of the interplay between ZEN and wheat physiology.

## Supporting information

S1 File **A Fig**: UHPLC-Q-Exactive full scan extracted ion chromatogram (resolving power 70,000 FWHM, extraction window 5 ppm) of ZEN-treated samples at 14 days in (A) leaves growing medium, in (B) roots growing medium compared to (C) matrix-matched calibration standard sample. **B Fig**: Full scan extracted ion chromatogram (resolving power 70,000 FWHM, extraction window 5 ppm) of control medium at (A) t = 0 and (B) after 7 days. ZEN R_t_: 12.16 min; cis-ZEN R_t_: 12.41 min. **C Fig**: Conversion of ZEN to cis-ZEN in the blank growing medium under the experimental conditions, over 2 weeks of observation. **D Fig**: Extracted ion chromatogram (resolving power 70,000 FWHM, extraction window 5 ppm) of control samples at 14 days: (A) control roots, (B) control leaves, (C) ZEN standard solution. **E Fig**: Main ZEN modified forms found in leaves (A) and roots (B). **F Fig**: Putative structure formula of ZEL-Sulf and characteristic sulfoconjugated fragment confirming the neutral loss of SO_3_. As a consequence, the intact ZEL molecule was observed (m/z 319.1562, [M-H]-). **G Fig**: LC-HRMS/MS characteristic fragmentation pattern and putative structure formula of ZEN-HexPent. Deprotonated adduct (m/z 611.2325) was fragmented with collision energy of 12 eV, highlighting characteristic loss of pentose (C_5_H_8_O_4_) and hexose (C_6_H_10_O_5_), showing the intact ZEN molecule (m/z 317.1376, [M-H]-).
(DOC)
