# Supplementary material for: Plant organ cultures as masked mycotoxin biofactories: Deciphering the fate of zearalenone in micropropagated durum wheat roots and leaves
Source: PLoS One. 2017 Nov 16;12(11):e0187247. doi: 10.1371/journal.pone.0187247 (PMC5690627; doi:10.1371/journal.pone.0187247)
Supplement: S1 File — A Fig: UHPLC-Q-Exactive full scan extracted ion chromatogram (resolving power 70,000 FWHM, extraction window 5 ppm) of ZEN-treated samples at 14 days in (A) leaves growing medium, in (B) roots growing medium compared to (C) matrix-matched calibration standard sample. B Fig: Full scan extracted ion chromatogram (resolving power 70,000 FWHM, extraction window 5 ppm) of control medium at (A) t = 0 and (B) after 7 days. ZEN Rt: 12.16 min; cis-ZEN Rt: 12.41 min. C Fig: Conversion of ZEN to cis-ZEN in the blank growing medium under the experimental conditions, over 2 weeks of observation. D Fig: Extracted ion chromatogram (resolving power 70,000 FWHM, extraction window 5 ppm) of control samples at 14 days: (A) control roots, (B) control leaves, (C) ZEN standard solution. E Fig: Main ZEN modified forms found in leaves (A) and roots (B). F Fig: Putative structure formula of ZEL-Sulf and characteristic sulfoconjugated fragment confirming the neutral loss of SO3. As a consequence, the intact ZEL molecule was observed (m/z 319.1562, [M-H]-). G Fig: LC-HRMS/MS characteristic fragmentation pattern and putative structure formula of ZEN-HexPent. Deprotonated adduct (m/z 611.2325) was fragmented with collision energy of 12 eV, highlighting characteristic loss of pentose (C5H8O4) and hexose (C6H10O5), showing the intact ZEN molecule (m/z 317.1376, [M-H]-). (DOC) [file pone.0187247.s001.doc]

**Supplementary Information**

**Plant organ cultures as masked mycotoxin biofactories: Deciphering the fate of zearalenone in micropropagated durum wheat roots and leaves.**

Laura Righetti^1^, Enrico Rolli^2^, Gianni Galaverna^1^, Michele Suman^3^, Renato Bruni^1^, Chiara Dall’Asta^1*^

^1^Department of Food and Drug, University of Parma, Viale delle Scienze 17/A, I-43124 Parma, Italy

^2^Deparment of Biosciences, University of Parma, Via G.P. Usberti 11/a, Parma, Italy

^3^ Barilla G.R. F.lli SpA, Advanced Laboratory Research, via Mantova 166, Parma, Italy

1. **Additional information on the Experimental Section**

**Sample preparation:** Plant samples were freeze dried for 24 h using a laboratory lyophylizator (LIO-5PDGT, 5Pascal s.r.l., Trezzano sul Naviglio, Milano) and then milled. 50mg of homogenized plant material were extracted by adding 1500 µL of solvent mixture of acetonitrile/water/formic acid (79:20:1, v/v) and stirred for 90 min at 200 strokes/min on a shaker. The extract was centrifuged for 10 min at 14000 rpm at room temperature, then 500 µL of supernatant were evaporated to dryness under nitrogen and finally reconstructed by 500 µL of water/methanol (80:20, v/v) prior to LC-MS analysis. All medium samples were diluted with water/methanol (80:20, v/v) to achieve a final ratio of 1:1 (v/v), vortexed for 1 min and then subjected to LC-MS analysis.

**UHPLC-HRMS analysis:** UHPLC Dionex Ultimate 3000 separation system coupled to a Q-ExactiveTM high resolution mass spectrometer (Thermo Scientific, Bremen, Germany) equipped with an electrospray source (ESI) was employed. For the chromatographic separation, a reversed-phase C18 Kinetex column (Phenomenex, Torrance, CA, USA) with 2.10×100 mm and a particle size of 2.6µm heated to 40 °C was used. 10 μl of sample extract was injected into the system; the flow rate was 0.4 ml/min. Gradient elution was performed by using 1 mM ammonium acetate in water (eluent A) and methanol (eluent B) both acidified with 0.5% acetic acid. Initial conditions were set at 10% B followed by a linear change to 40% B in 4 min and to 90% B in 16 min. Column was then washed for 2 min with 90% B followed by a reconditioning step for 3 min using initial composition of mobile phases. The total run time was 25 min. The Q-Exactive mass analyzer was operated in the full MS/data dependent MS/MS mode (full MS–dd-MS/MS) at following parameters: sheath and auxiliary gas flow rates 32 and 7 arbitrary units, respectively; spray voltage 3.3 kV; heater temperature 220 °C; capillary temperature 250 °C, and S-lens RF level 60. Following parameters were used in full MS mode: resolution 70,000 FWHM (defined for m/z 200; 3 Hz), scan range 100–1000 m/z, automatic gain control (AGC) target 3e6, maximum inject time (IT) 200 ms. Parameters for dd-MS/MS mode: intensity threshold 1e4, resolution 17,500 FWHM (defined for m/z 200; 12 Hz), scan range 50 – fragmented mass m/z (m/z +25), AGC target 2e5, maximum IT 50 ms, normalized collision energy (NCE) 35% with ±25% step.

**Putative identification of ZEN metabolites:** The full identification of ZEN, α-ZEL, and β-ZEL was obtained by comparison with commercial standards. Similarly, *cis*ZEN, ZEN14Sulf, ZEN14Glc, and ZEN16Glc were accurately identified by comparison with authentic standards, obtained by chemical or enzymatic synthesis (Köppen et al. 2012; Dall’Erta et al. 2013; Kovalski-Paris et al. 2014). For other metabolites, the annotation process involved the following items; (i) the measured accurate mass of [M-H]¯ must fit the theoretical accurate mass with a mass tolerance set at ±5 ppm, (ii) isotopic pattern: the experimental and theoretical isotopic patterns shall correspond, (iii) MS-MS spectra: product ion of intact ZEN (m/z 317.1389) and ZOL (m/z 319.1550) and/or comparison of the fragments obtained with the fragmentation pathway of ZEN or other mycotoxins metabolites formerly found (Berthiller et al. 2006; Meng-Reiterer et al. 2015; Meng-Reiterer et al. 2016). Only in few cases, fragmentation spectra could not be collected, due to parent ion abundance below the threshold. In this case, a tentative annotation based on accurate mass and elemental formula was performed, as already proposed by other authors (Meng-Reiterer et al. 2015; Meng-Reiterer et al. 2016)

The annotation process followed within this study, has been recently described by the authors. See Righetti et al (2016) for full details.

Examples of spectra used for metabolite identification are reported in Figure 5S and 6S.

**Statistical analysis**

All statistical analyses were performed using IBM SPSS v.23.0 (SPSS Italia, Bologna, Italy). Data were analysed by Kruskal-Wallis test followed by Duncan post-hoc test (α = 0.05).

1. **Additional information on the Results Section**

*Occurrence of ZEN metabolites in the growing media*

To monitor the evolution of ZEN and the possible secretion of masked mycotoxins, medium samples were taken (in aseptic condition) from both root and leaf culture at the start of experiment (t0), then after 1, 6, 12, 24 hours, 7 and 14 days and kept refrigerated until analysis. All medium samples were diluted with water/methanol (80:20, v/v) to achieve a final ratio of 1:1 (v/v), vortexed for 1 min and then subjected to LC-MS analysis.

Modified forms of ZEN were never detected in the growing medium, as reported in Figure 1S. A partial isomerization of ZEN to cis-ZEN was detected in the growing mediaum, after 7 and 14 days of treatment, reaching a 40:60 *trans:cis* ratio at 14 days, as reported in Figure 2S and in Figure 3S.

*Occurrence of ZEN metabolites in roots and leaves*

In order to avoid bias, the occurrence of ZEN metabolites in roots and leaves at 14 days was compared with results recorded at time 0, and with those obtained for control plant material (14 days, no treatment). Data obtained at 14 days are reported in Figure 4S.

The chromatograms of the main compounds identified in leaves and roots after 14 days, are reported in Figure 5S.

**Figure A:** UHPLC-Q-Exactive full scan extracted ion chromatogram (resolving power 70,000 FWHM, extraction window 5 ppm) of ZEN-treated samples at 14 days in (A) leaves growing medium, in (B) roots growing medium compared to (C ) matrix-matched calibration standard sample.


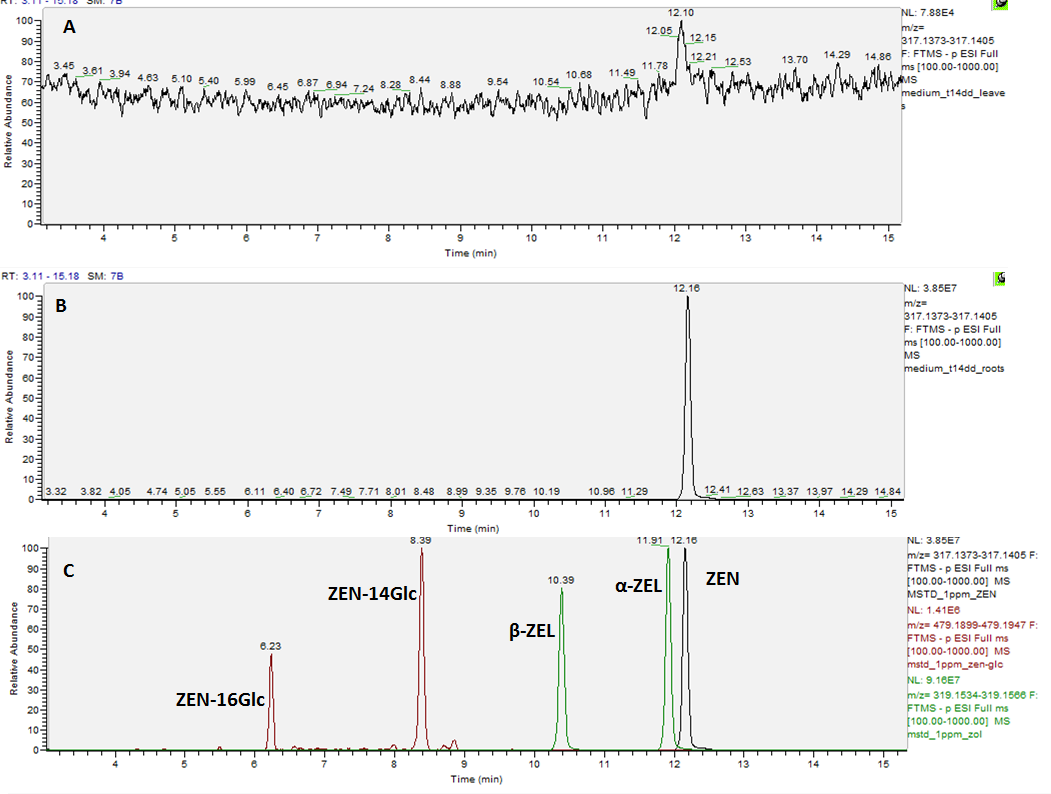


**Figure B:** Full scan extracted ion chromatogram (resolving power 70,000 FWHM, extraction window 5 ppm) of control medium at (A) t = 0 and (B) after 7 days. ZEN R_t_: 12.16 min; cis-ZEN R_t_: 12.41 min.


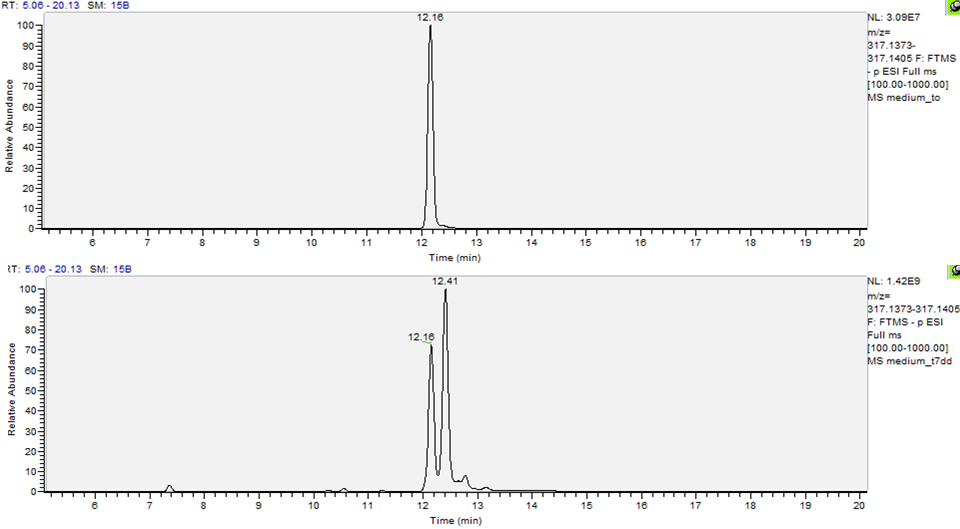


**Figure C:** Conversion of ZEN to cis-ZEN in the blank growing medium under the experimental conditions, over 2 weeks of observation.


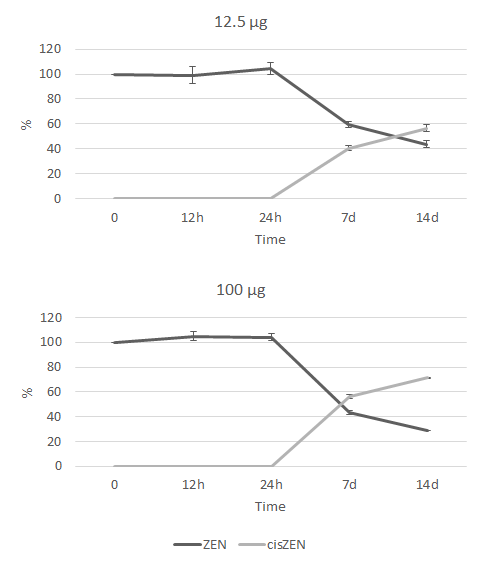


**Figure D**: Extracted ion chromatogram (resolving power 70,000 FWHM, extraction window 5 ppm) of control samples at 14 days: (A) control roots, (B) control leaves, (C) ZEN standard solution.


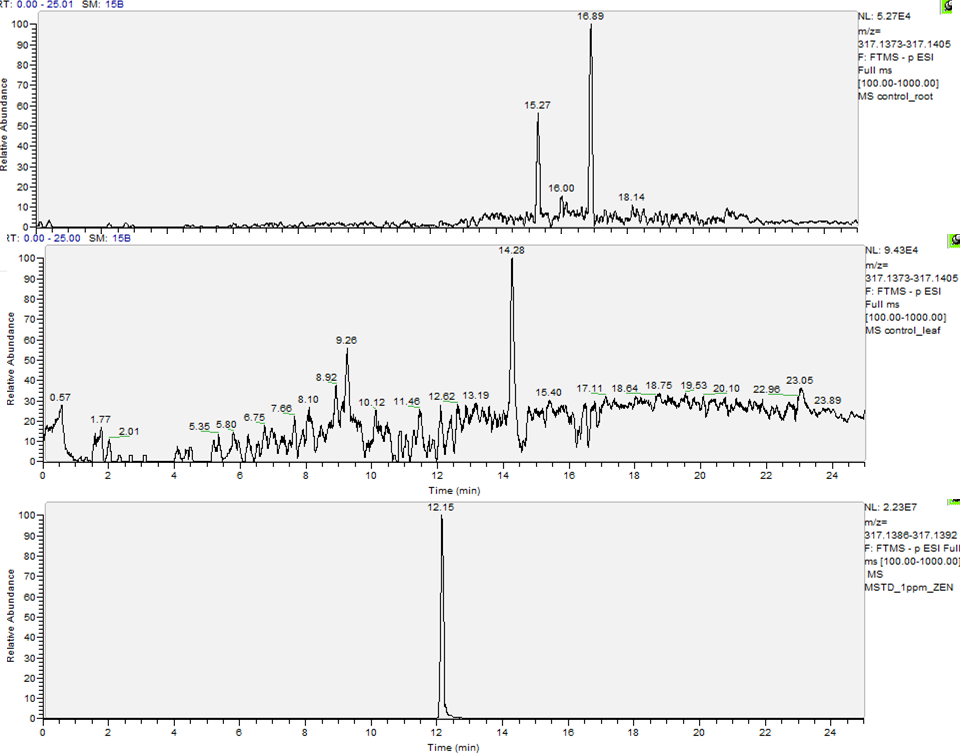


**(A)**

**(B)**

**(C)**

**Figure E**: Main ZEN modified forms found in leaves (A) and roots (B).


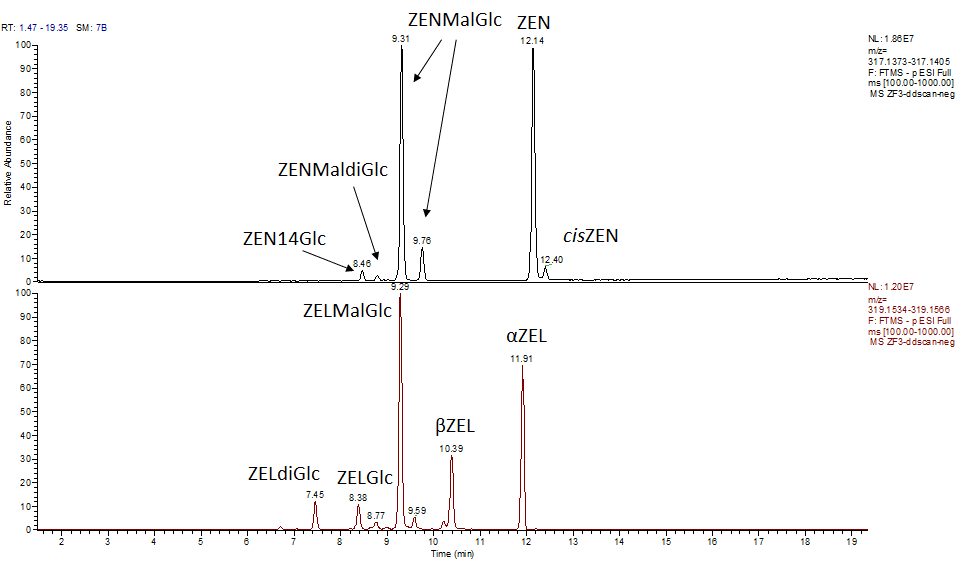


**(A)**

**(B)**

**Figure F:** Putative structure formula of ZEL-Sulf and characteristic sulfoconjugated fragment confirming the neutral loss of SO_3_. As a consequence, the intact ZEL molecule was observed (m/z 319.1562, [M-H]-).


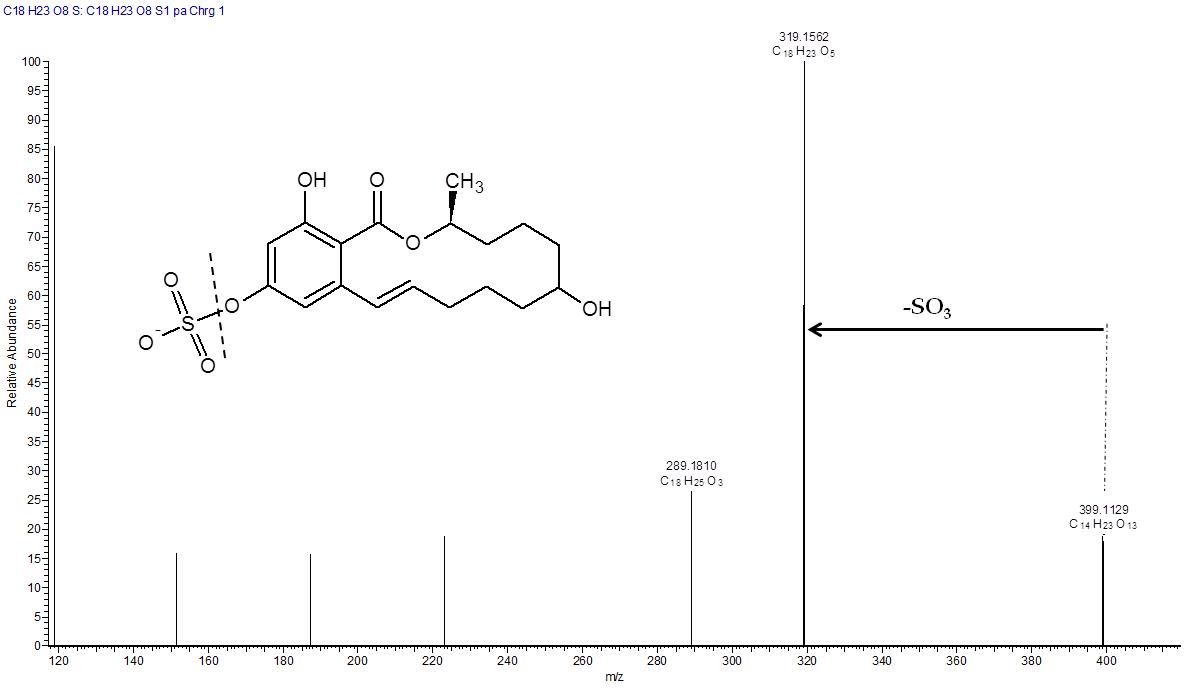


**Figure G:** LC-HRMS/MS characteristic fragmentation pattern and putative structure formula of ZEN-HexPent. Deprotonated adduct (m/z 611.2325) was fragmented with collision energy of 12 eV, highlighting characteristic loss of pentose (C_5_H_8_O_4_) and hexose (C_6_H_10_O_5_), showing the intact ZEN molecule (m/z 317.1376, [M-H]-).

**
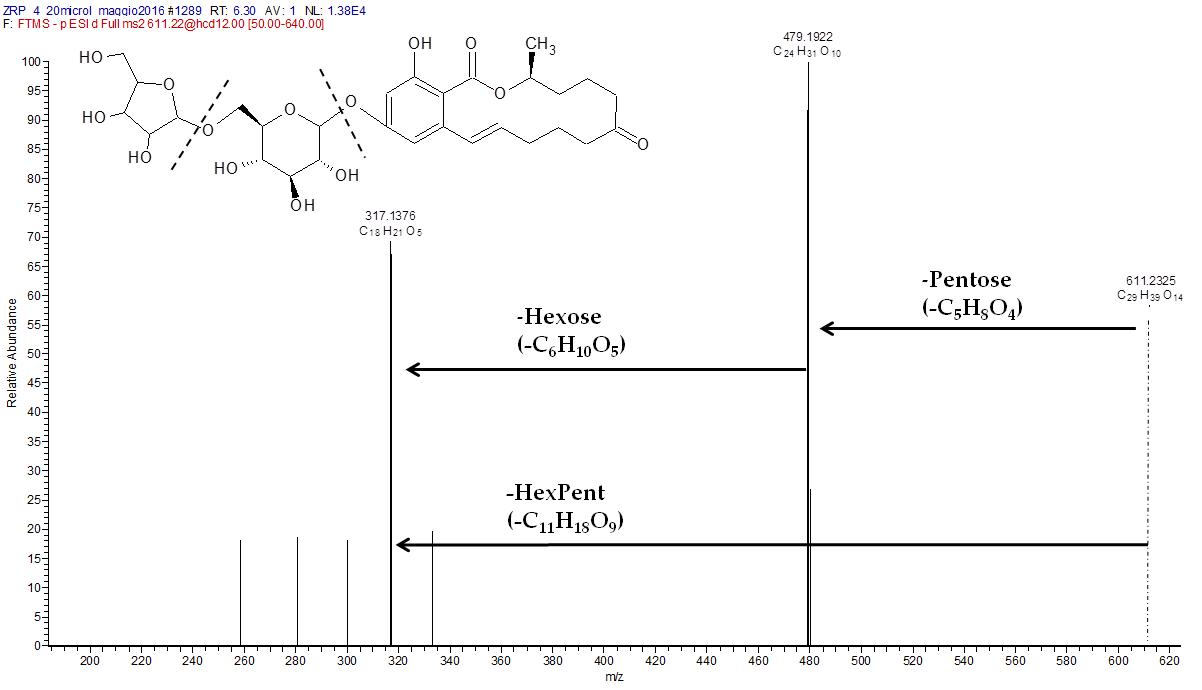
**

**References**

- Dall’Erta, A., Cirlini, M., Dall’Asta, M., Del Rio, D., Galaverna, G., & Dall’Asta, C. Masked mycotoxins are efficiently hydrolyzed by human colonic microbiota releasing their aglycones. *Chem. Res. Toxicol.* **26**, 305-312. DOI 10.1021/tx300438c (2013).
- Köppen, R., et al. Photochemical trans-/cis-isomerization and quantitation of zearalenone in edible oils. *J. Agric. Food Chem.* **60**, 11733-11740. DOI 10.1021/jf3037775 (2012).
- Kovalsky Paris, M. P., et al. Zearalenone-16-O-glucoside: a new masked mycotoxin. *J. Agric. Food Chem.* **62**, 1181-1189. DOI 10.1021/jf405627d (2014).
- Berthiller, F., Werner, U., Sulyok, M., Krska, R., Hauser, M.T. and Schuhmacher, R. Liquid chromatography coupled to tandem mass spectrometry (LC-MS/MS) determination of phase II metabolites of the mycotoxin zearalenone in the model plant Arabidopsis thaliana. Food Addit Contam. 23, 1194–1200. DOI 10.1080/02652030600778728 (2006).
- Meng-Reiterer J., et al. Metabolism of HT-2 Toxin and T-2 Toxin in Oats. Toxins, 8, 364-385; doi:10.3390/toxins8120364 (2016)
- Meng-Reiterer, J., et al. Tracing the metabolism of HT-2 toxin and T-2 toxin in barley by isotope-assisted untargeted screening and quantitative LC-HRMS analysis. Anal. Bioanal. Chem. 407, 8019-8033. DOI 10.1007/s00216-015-8975-9 (2015)
